# Medical Education and State Examinations for License

**Published:** 1898-03

**Authors:** William Warren Potter

**Affiliations:** President New York State Medical Examining and Licensing Board


### Progress in Medical Science.

### MEDICAL EDUCATION AND STATE EXAMINATIONS FOR LICENSE.

By WILLIAM WARREN POTTER, M. D.,
President New York State Medical Examining and Licensing Board.

### STATE MEDICAL SOCIETIES AND THE EXAMINING BOARDS.

DR. GEORGE BEN JOHNSTON, of Richmond, in his presidential address before the Medical Society of the State of Virginia, September, 1897, said: The creation of legal requirements to enter into the practice of medicine, accomplished by the various state societies acting independently of each other, has made a profound impression on the medical schools all over the country. Without exception they have advanced their requirements for entrance, prolonged their terms of study, and perfected their methods of teaching. They have also entered into hearty coöperation with the licensing boards and have banded themselves into a league whose purpose it is to still further elevate the standard of education and secure uniform methods of teaching. The natural consequence of these distinct advances and the attraction of public attention to the work in this line of societies and licensing boards brought about the formation of the Confederation of national examining and licensing boards. This organisation is yet young but rapidly pushing forward to accomplish a splendid work, and it deserves the hearty support and coöperation of every state society and licensing board. Its objects are to exalt medical education, to establish a fixed standard of requirements to enter the profession of medicine, to procure uniformity of laws regulating the practice of medicine, and reciprocity between the various state organisations by means of which the license of one board will be recognised and accepted everywhere. These are aims greatly to be desired, and from the present trend of legislation it is not too much to hope that they will be speedily accomplished. There is no need for a national board of licensers. All of this should be regulated by the representatives of the various states assembled in the councils of the national confederation agreeing upon definite methods and prerequisites.

---

### OHIO REGISTRATION AND EXAMINATION LAW.

THE Supreme Court of Ohio, (*Lancet-Clinic*, October 30, 1897,) by a unanimous decision rendered by its members, has declared the law to be constitutional.

This is a valuable victory for the medical profession, and will greatly simplify medical matters for the future.

In the above decision the case and cause of one Franz, of malodorous fame, was lost by the defendant, and he will be obliged to hie himself to the environs of other states for the purpose of conducting his quackery business.

The Ohio state board of registration and examination may now take courage and fearlessly go ahead in a prosecution of those who do not meet the requirements of the law. To the writer it appears to be quite apparent that the public is in sympathy with the physicians in their purification cause. The work is only begun and is very far from being ended. The board has the authority of the law to say what educational schools are in good standing. This will make an end of osteopathy, christian science, and all of that train of wickedness, including the claims of the hygeia medical college people of Cincinnati.

All is well that ends well.

### THE MARYLAND MEDICAL EXAMINING BOARD.

THE result of the state board medical examination last May (*Maryland Med. Jour.*, December 25, 1897,) and the unfortunate overthrow of so many candidates at that time caused so much dissatisfaction among the medical schools, that after several conferences between representatives of the various schools of Baltimore and some correspondence between a committee from the schools and the examining board, a meeting was called last week for the purpose of considering amendments to the medical practice act.

So well attended was this meeting that comment is scarcely necessary. Rather than a conference, it was more like a battle between those supporting the schools and those in sympathy with the board. The object of the proposed amendments was, among other things, to put members of teaching bodies on the examining board and compel examinations in the primary branches to be conducted by those who were teachers in the schools.

These amendments were proposed by the schools because it seemed that the board was unnecessarily severe on the graduates of schools, and students who had stood well during the term and had passed a creditable examination were turned down by men who were said to be inexperienced in conducting examinations and who were supposed not to be well versed in the elementary branches. There were some suggestions in the amendments that were undoubtedly good, but the amendment, as a whole, was voted down by a large majority, many men in the schools voting to support the board in its present course.

The objections to putting on the board men connected with schools are obvious and, as one of the board said, of the twenty-eight states that require a board examination, not one contains members from the teaching bodies. The dishonesty of candidates was to be deplored and perhaps a larger number than usual was caught in the act of breaking the pledge at this examination because previous tests had not been so strict.

It was, perhaps, a surprise to the college men that the board carried the day at this meeting, but the unbiased spectator cannot help seeing that the results of this meeting will be of advantage to both parties. The schools will be compelled to turn out a better set of men, more efficiently prepared for the final test, while the board itself will conduct examinations in such a way that the temptation to cheat will not be so great, and, also, even without compulsion, they will undoubtedly modify some of the rules which have governed them heretofore.

The board was appointed by the state faculty and is in a measure accountable to the faculty and when it makes up its report at the next annual meeting, perhaps it would be well to clearly explain all its actions without the vague phraseology that occurred in its reply to the committee of the colleges. The board is above all things honest and sincere in its work, as its members have no other motive than to do their duty and certainly they have done it well.

It is very unfortunate that the board has no power to drive out illegal practitioners, but the law gives it power to examine and license only, while the privilege of detecting is left to any member to whose notice any irregularities may come. It is probably better for the community and the cause of higher education that the amendments were defeated.

---

### NONGRADUATE APPLICANTS IN ILLINOIS.

THE Illinois state board of health (*Medical Standard*), at the quarterly session held in Chicago on October 5th, adopted the following resolution with respect to the examination of nongraduates applying for certificates to practise medicine :

*Resolved,* That after May 1, 1898, all nongraduate applicants for license to practise medicine and surgery, who are examined in accordance with the provisions of the medical practice act, in addition to the requirements already exacted, must present as evidence of a satisfactory preliminary education, either :

1. A diploma or certificate of graduation from a high school.

2. A certificate of having passed the matriculation examination to a recognised literary or scientific college.

3. A certificate of successful examination by the faculty of any reputable university or college of arts or science (not members of a medical college faculty), by the state superintendent of public instruction of Illinois or by the principal of a high school in Illinois, in the following branches : English grammar, arithmetic, elementary physics, United States history, geography and Latin equivalent to one year in a high school.

Each candidate will alse be required to present a certificate from a medical college in good standing with this board, attesting that the applicant has—

1. Pursued the study of practical anatomy in said college for at least one term and has made dissections of the entire cadaver.

2. Taken at least one full course in operative surgery and practical obstetrics.

3. Personally attended six or more cases of labor.

Bacteriology has been added to the subjects of the nongraduate examination.

In taking this step the board has acted wisely and entirely within the scope of its authority. The Illinois statute governing the practice of medicine does not require that applicants shall be graduates of a medical college, but it does assume that persons presenting themselves for examination shall be possessed of those qualifications which common law has established as customary for the discharge of such important public duties as constitute the practice of medicine. This standard of qualification, however, is not and never has been a fixed one, or even a minimum one. The standard owes its degree of minimum requirements to the action of the body of the institutions teaching medicine. The medical colleges create the standard, and as facilities for imparting scientific knowledge multiply, the standard is constantly advanced. Recently the Illinois medical colleges have announced requirements regarding a preliminary education as a condition for admission, and the state board of health is justified, in the spirit of the law governing its actions, in advancing its requirements to accord with the prevailing standard established by the colleges.

In adding bacteriology to the subjects of the nongraduate examination, the board properly recognises a department of medical science now imperative in medical knowledge.

The board incidentally reaffirms its position with reference to medical colleges in "good standing" and leaves to those institutions the same authority in regard to preliminary educational requirements of their matriculants as they have exercised heretofore. The law seems fully established and generally understood by boards that in the absence of statutory authority to prescribe minimum entrance requirements for colleges, the extent of the authority of the board is limited to an act of approval and does not contemplate mandatory rules. The decision rendered by the Missouri court adverse to the action of the Missouri board of health in issuing minimum matriculation requirements is clear on this point.

The action taken by the Illinois board will be approved by those who supported the effort to have a new medical practice act passed by the last legislature. The proposed law had two fundamental propositions: first, that no certificate should be issued to practise medicine in the state without examination, and, second, that a diploma from a medical school approved by the board should be a requisite to entitle to examination. In the new rule formulated the board has advanced toward this standard as far as the text and spirit of the existing law authorises.

---

### CENTER OF MEDICAL EDUCATION.

A GRADUAL change is taking place in the center of medical education. Comparing the statistics of 1888–1889 with those of 1894–1895, we find that the Chicago medical institutions have increased in attendance from 1,338 to 2,294, while the New York medical colleges show a decrease in attendance, which has fallen from 2,081 to 1,893. Baltimore has almost doubled its attendance and now has 1,293 students. St. Louis now takes fourth place, having passed Baltimore, Cincinnati and Louisville, and it now stands fourth, with an attendance of 1,399, while Louisville is sixth, with 947 students, a decrease of 43. Philadelphia, which was over 500 behind New York in the first period, is now over 300 ahead, with an attendance of 2,201, a gain of 686 over the figures of 1888–89.—*Journal A. M. A.*

A very natural question regarding the above is, why have such changes taken place? New York being the largest city in the union, we would expect her to offer better inducements and have a larger medical class than other smaller cities. If one studies a few facts concerning the case, possibly an explanation may be forthcoming. It will be remembered that New York state has taken the lead in the last six or ten years in raising the standard of medical education. She was the first to require as a prerequisite to the study of medicine a literary education to some degree above the high school. A state law was passed later raising the standard higher and requiring all who did not have a college degree to pass a satisfactory examination before a state board. At the same time the medical colleges in the city of New York increased their course from two to three, then to four years, and also enforced harder final examinations. Medical colleges in other cities have followed suit, but all medical colleges in each city did not, many of them remaining more or less lax in their requirements for several years and many of them still so; especially is this true of St. Louis, which shows the largest proportionate gain.

Possibly, indeed probably, these facts account for the changes in the number of students studying in the various cities. Medical students, as a rule, are apt to go the way that offers the least resistance.—*S. W. Med. and Surg. Reporter.*

### KANSAS NEEDS A PRACTICE LAW.

What is needed in Kansas (*Kansas City Med. Record*) and in many other states is a stringent law regulating the practice of medicine. It is somewhat singular that other laws seem easy of enactment in Kansas, while a law to protect the people from empirics cannot be passed. We believe this is to some extent the fault of the profession generally. Some hold the view that these matters will regulate themselves, but observation teaches us that they do not. Again, others argue that there is no use to make an effort, as the legislature will not heed the demands. We believe this is an error. Qualified physicians in the state of Kansas are sufficiently powerful to cause the enactment of any just and reasonable law which they may agree upon. The action should not be delayed till after the legislature meets, but should be commenced as soon as the legislature is elected. Every physician in the state should constitute himself a committee of one to look after the legislators of his acquaintance, and instruct them fully in regard to the matter, and there will be no trouble in passing any law that is just and equitable.

---

### CONSTITUTIONALITY OF LAWS REGULATING PRACTICE OF MEDICINE.

Under the civil law (*Jour. of Med. and Science*) no barriers were drawn around either professions of law or medicine. Anyone who pleased might practice them without any previous qualification, subject always for injuries inflicted upon others. The common law, having an habitual regard for the widest freedom of action and borrowing from the civil law, always favored the right of any man to practise in any profession or business in which he was competent. It was a right inherent in every man to practise the art of medicine. But in modern times the tendency of legislation is to prescribe qualifications for the practice of the professions of law and medicine. Its competency cannot be successfully impugned. Learning and good moral character may be required as a condition to the exercise of the rights to practise them. A law enjoining reasonable requirements as to learning and character is simply an exercise of the police power. This power may be exercised in barring from admission those who apply for the right to practise the profession of medicine and also in excluding those who have been practising it. Such legislation is justified by the safety of the lives, health and comfort of the people, which is the supreme law. In no case is the practice of medicine a property right. Nor is it vested right. But it is a valuable right and no one can arbitrarily and without reason be deprived of it. Such is the general statement made by Judge Pugh, of the court of common pleas of Franklin county, Ohio, in the case of State vs. Ottman, where he holds the law of that state passed February, 1896, entitled "An act to regulate the practice of medicine," constitutional. That it authorises the medical board to refuse to grant or revoke a certificate to practise "if any person guilty of felony or gross immorality," he does not consider in violation of the provision of the constitution of the United States which prohibits the passage of any ex-post facto law or bill of attainder, though it was argued that this was a deprivation of a civil right for past conduct. Neither does he think that it violates the provision which ordains that the citizens of each state shall be entitled to all privileges and immunities of citizens of the several states; nor anything in the fourteenth amendment; nor the constitution of the state, especially in its provisions as to appeals.

---

### PHYSICIANS AS POLITICIANS.

THE need of organisation and the value of it, too, (*Maryland Medical Journal*, January 8, 1898,) have become more apparent to the medical profession in recent years. Physicians, as a rule, act in harmony in their scientific deliberations, but when it comes to a question of proposing and enacting laws which are for the benefit of the people as well as for the profession, the latter are as babes in arms. This weakness is especially noticeable at each session of the Maryland legislature.

The most important questions pertaining to the health and welfare of the people are presented to the legislature in the most emphatic manner by a large delegation of really prominent physicians, and notwithstanding facts are clearly stated the whole matter is lost by the political influence of one or two opponents, simply because prominent physicians with no "pull" have no weight with politicians.

An example of this was in the early attempts to pass a medical practice act in Maryland, which at first was easily defeated by influence. And even now, when the medical law licensing the physician and protecting the public of Maryland is in a fairly good shape, it is stated that influence will be used at the coming meeting of the legislature to make serious changes and actually weaken the effect of the law; and this in spite of the fact that the state faculty, which is, or should be, behind every medical question, voted by a large majority to leave this act unchanged.

It is in just such questions as these that the better class of physicians should recognise the fact that the profession should know something of practical politics and thus meet their enemies with their own weapons. As was stated before, twenty-eight states have medical laws and no single board contains members from one of the medical schools in that state. Why should Maryland be an exception? Is it a confession of weakness on the part of the schools, or is there some other reason for this desired change? The members of the examining board certainly have nothing to gain or lose by passing or rejecting candidates. They are certainly sincere in their work.

On the other hand, they have been criticised, and justly criticised, too, for asking questions of an unpractical nature, some of which probably no physician could answer. Because the examining board is not infallible and makes mistakes, it would be very foolish for the profession of the state to make matters worse by making an additional mistake and thus weakening a good law.

The examining board should report to the faculty at its next annual meeting and should not be too high and mighty to explain its acts and receive advice as to the kind of questions to ask and the manner of conducting the examination. It should be the duty of every physician of Maryland, who desires a high standing of his profession, to give his views on this important question without that quibbling and false reasoning which was so apparent at the recent special meeting of the faculty.

To show the feeling of some of the members, one physician present at that meeting said he was heartily in favor of sustaining the examining board and he was against any proposed change in the law, but as a member of the faculty of one of the schools he felt obliged, and in reality had promised, to vote against his convictions. Such a feeling will hardly tend to elevate medicine.

One danger of bringing this question before the legislature is that ignorant members may make further amendments and entirely destroy the value of the whole act. If this question does come before the legislature it will be a question of influence rather than of merit.

### ENFORCING THE OHIO MEDICAL LAW.

The Hamilton County Prosecutor is busy. Within the last few days (*Lancet-Clinic*, January 22, 1898,) there have been more than twenty indictments found by the grand jury against those who are engaged in practising medicine illegally. One or more are in jail, unable to give a small bond. Those who are indicted have against them a sufficient proof to warrant a belief that they will be convicted.

The good work will go on until the people are relieved of the presence of a class of persons whose very recognition is a menace to the ignorant and a readiness to become copartners in guilt with those who, in the eyes of the law, are engaged in criminal occupations. The Ohio law is an effective instrumentality, and as such has been declared by the State Supreme Court to be constitutional.

---

### THE ALABAMA SYSTEM.

We publish in another column (*Western Medical Review*, Febru-15, 1898,) a paper on the Alabama system. It will surprise many of our readers to know that any state in the union had such an excellent law. It seems that in this regard the South is far ahead of the North. Mississippi's law is very similar to that of Alabama, except that the former has but one board of examiners, which is also the board of health. This board consists of twelve members, and is appointed by the governor, five of whom, however, must be appointed upon the recommendation of the state medical society. The law in both of these states is faulty in that neither has the power to revoke certificates.

The idea of having county examining boards, at first thought, would seem to be bad, but the proof of the pudding is in the eating, and it proves, after nearly twenty years' trial, to be all right. It certainly has done one good work for each county ; it has brought about the organisation of a live, active, working county medical society in every county in the state. Dr. Sanders, in the discussion following the reading of the paper, said : "The organisation of a county medical society in every county in my state I regard as an achievement of very great value and as far outweighing any temporary evil that may have resulted from granting licenses to a few incompetent men." We would urge the reading of Dr. Sanders's paper and would call attention to the way our Alabama friends get rid of the sectarian bugaboo.

[Dr. Sanders's paper in full will be found in the Transactions of the National Confederation of State Medical Examining and Licensing Boards for 1897, also published in the Bulletin Am. Acad. Med., December, 1897.—*Compiler.*]

---

### COMPULSORY REGISTRATION REFUSED.

JUDGE HOLMES, in the Massachusetts supreme court, (*Boston Med. and Surg. Jour.*, Jan 20, 1898,) recently dismissed the petition brought by Michael Nolan, of Haverhill, asking for a writ of mandamus to compel the board of registration in medicine to give him a certificate of registration. Nolan applied to the Board on December 10, 1894, setting forth in his application that he was a graduate of Vermont Medical College, at Rutland, in 1889, and that he had practised four years in Haverhill. In his petition to the court he claimed that he was a graduate of a legally chartered college having power to confer degrees in medicine and therefore wrongly denied registration. The Board in its reply to the declarations of the petitioner asserted that the college named was a fraudulent institution and under the laws of Vermont was not authorised to confer medical degrees ; the judge ruled accordingly.

